# Round the Hospitals

**Published:** 1917-11-03

**Authors:** 


### ROUND THE HOSPITALS.

The Prime Minister, in the course of his tribute to the officers, petty officers, and men of the Navy, the officers, non-commissioned officers, and men of the British Armies in the field, and also to the women in the Medical and other Services auxiliary thereto, the gallant troops from the Dominions overseas, from India, and from the Crown Colonies, the officers and men of the Mercantile Marine, and the Houses of Parliament's acknowledgments, with grateful admiration, of the valour and devotion of those who have offered their lives in the service of their country, spoke as follows: "We should also thank the women, our trained and untrained nurses, whose tenderness and care for the wounded have earned thanks from the lips of hundreds of thousands of poor men, whose lives have been saved and who have been spared much suffering through their tender ministration. They have not escaped perils. Many have been killed by shell-fire, many of them drowned in hospital ships, sunk with the sign of the cross upon them. We all owe them a debt of gratitude, and also to the men of the Royal Army Medical Corps and the Army Service Corps, who were all in the firing-line and have suffered very severely." Mr. Asquith, responding to a request by Mr. Lloyd George, in the course of his speech mentioned: "Our doctors and nurses, our fellow countrymen and countrywomen, enlisted in every department of war work, who from all quarters of the Empire have by their ceaseless energy and unbounded sacrifice ensured the victory of the Allies. Among all these comrades and fellow-combatants, for such they are, in the greatest of causes, we make no discrimination in the degree of our gratitude. It is by their united efforts and unextinguishable faith that the struggle has been, and will be, maintained till it ends, as we know it must end, in the enthronement of the sovereignty of right."

---

Few hospital workers at home have any real conception of the actual life and work devolving

**ROUND THE HOSPITALS**—*(continued).*

upon nurses employed in France who are attached to hut and tent hospitals, clearing stations, and elsewhere, where they are in constant attendance upon the wounded from the fighting-lines. Sisters and nurses thus employed are liable at any moment to be called upon to face serious danger and to handle cases of the most trying and sometimes of the most appalling character. We hear something of the bravery and heroism of British nurses, but observation and experience at the Front convince us that most of the heroic deeds occur in circumstances which do not lend themselves to publicity, nor is such a thought ever in the minds of the heroines or heroes, or of those who know the facts.

Take the case of employment in a tent hospital which is liable to continuous raids at any time, where there is absolutely no shelter or protection available worthy of the name. Do the nurses ever seek work elsewhere? Our experience justifies the answer, Never. Indeed, the more competent and highly trained the nurse, the more certain is it that she will resist any suggestion or alteration of regulations which puts her in a place of safety when the adoption of this course separates her from her patients. The spirit of Florence Nightingale is mercifully prevalent throughout the nursing ranks at the seat of war, and our nurses refuse absolutely to leave their charges, preferring to share whatever danger there may be with their patients who are unable to help themselves, and who are dependent upon the nurse's services in the case of disaster or further disablement. Could anything be written more likely to stir up the hearts and consciences of British men and women who remain at home to the splendid privilege which each one of them is offered by the founding of the Nation's Fund for Nurses? which of them all will refuse to help the British Women's Hospital Committee speedily to bank the capital necessary to provide sufficient funds to enable the new departure, to help the nurses to help themselves, to become a permanent and ever-growing success? War-time is not a time for talking, much less for gossip. It is the hour for action, and everybody who has the spirit of nationality and the love of God in their hearts will not go to bed until they have sent their contribution to the Nation's Fund for Nurses as an act of thanksgiving, and a just recognition of splendid services rendered.

The official *Gazette* of October 25, 1917, has an interest for all matrons, sisters, and members of the profession. It contains nearly three columns of closely printed names, being ladies of the Nursing Service, to whom His Majesty the King has been pleased to award the Royal Red Cross decoration in recognition of their valuable services in connection with the war. The *London Gazette* is not regarded as a humorous newspaper, but the pressure of war-time has compelled the inclusion in its columns of the announcement in this connection: "Each lady is Miss unless otherwise stated." The publication of these few words, simple as they are, may be attended with far-reaching consequences, the effects of which we hope to live to have the pleasure of recording, with our congratulations.

It is estimated that altogether something like 1,000 members of the British nursing profession of all ranks are included in the *Gazette's* notice of the 24th ultimo. Of these, about one in ten has received the Royal Red Cross decoration of the First Class, and the remainder have been awarded the Second Class of the same Order. In this estimate no allowance has been made for the V.A.D.s, being commandants, who have received the Second Class of the Royal Red Cross. A grateful feature of the notices in the *Gazette* is the fact that amongst the names is that of a retired matron, Miss M. A. Girdlestone, who, by devoted service and sheer ability, during the best years of her life, has won an undying reputation in connection with the great services she has rendered to Poor-Law nursing, especially in connection with the Crumpsall Infirmary, Manchester. The action of His Majesty shows how searching is the Royal eye when merit is the quarry, and the inclusion of Miss Girdlestone's name cannot fail to give encouragement and genuine pleasure to all who have the best interests of the nursing profession at heart.

The leading Journal's advertisements on October 29 included a whole-column appeal for the Nation's Fund for Nurses. It contained no allusion whatever to the importance and vital interests underlying the work of the British Women's Hospital Committee in the direction of hastening the immediate development of a central organisation, representative of the nurses, which can authoritatively speak in their name and become sufficiently powerful to compel attention to the views of British-trained nurses throughout the Empire. The College of Nursing will provide the British nursing profession with a central organisation in the hands of British nurses, with the aid of those who possess the experience and up-to-date knowledge, and the unselfish devotion to secure everything that is best for the individual nurse in the pursuit of her profession. Progress has been made by the College already in certain directions, but the time has come when, with the support of the nation, its influence, radiating from its London, Scottish, and Irish centres, must quickly spread through the United Kingdom by means of affiliated branches, and thence awaken the Empire, wherever trained nurses are engaged in ministering to the sick.

The aims of the College are most important, and, when associated, as they must be, with the establishment of a recognised standard of education and training for the nursing profession, and the protection of the public against the half-trained or worthless woman, there is not a household, however humble or exalted in the land, the occupants of which will not necessarily, by their interest in these aims, gratefully make a point of contributing immediately a definite sum to the Nation's Fund for Nurses. We hope, therefore,

**ROUND THE HOSPITALS**—*(continued)*.

to see these important and pressing aspects of the British Women's Hospital Committee's scheme shortly formulated in the press, that no time may be lost in giving the widest opportunity to all the interests concerned, to show their appreciation of the British nurse by sending a liberal contribution to Lady Cowdray at 16 Carlton House Terrace, S.W. 1, without delay. Meanwhile we hope Lady Cowdray is receiving substantial sums for the second branch of the British Women's scheme for our nurses, that the College may be enabled to make provision for those of its members who through no fault of their own are in need or suffering. In this way the public can make some small return to the nurses, in discharge of the immeasurable debt all members of the community owe to the nurse, for her devoted ministrations to, and successful treatment of, the sick in the hour of their greatest need.

Her Majesty Queen Alexandra has been graciously pleased to appoint Miss Amy Hughes, late General Superintendent of Queen Victoria's Jubilee Institute for Nurses, as a member of the Council of the Institute. We consider this appointment of supreme importance, seeing that Miss Hughes is properly regarded as a leader in nursing affairs, an able administrator, and possessing the power of doing many things which press, if the College of Nursing is rapidly to assume the commanding position which it ought to secure, in the best interests of British nurses throughout the Empire.

The British Women's Hospital Committee have issued their appeal for a Nation's Fund for Nurses. That appeal in various forms has appeared in the advertising columns of the *Times*, the *Daily Telegraph*, the *Evening Standard*, and the *Observer*. The *Spectator* and the *Observer* have shown marked interest in the scheme, and have backed it by forcible articles in consecutive issues, which is very encouraging and helpful. The *Manchester Guardian* and the *Liverpool Courier* have each given substantial support to the Nation's Fund for Nurses, and the *Scotsman* has published the appeal of the British Women's Hospital Committee. In thanking our contemporaries for their invaluable help in the cause of the nurses, we hope to receive, as they appear, copies of other papers containing articles on the Nation's Fund for Nurses, together with reprints of the advertisements, letters, and other contributions in aid of the Fund. We ask this to enable us to keep the nurses informed of their friends who are working for them in the press of the British nation. Full particulars of the Nation's Fund and its objects may be obtained on application to the British Women's Hospital Committee, 21 Old Bond Street, London, W. 1.

There is already one important and encouraging outcome of the action taken by the British Women's Hospital Committee on behalf of British nurses. An officer in the British Army, with others, has addressed himself to the question of the pay of British nurses, which he properly holds is at present inadequate. He is most anxious to ascertain if anyone is taking action with the view to removing a state of things which reflects adversely on the reputation of the British public, who have given such splendid instances of spontaneous generosity since the outbreak of the Great War. We welcome this spontaneous interest in the remuneration of British nurses of all grades, and believe that one indirect but important outcome of the establishment of the Nation's Fund for British Nurses will be to cause independent action on the part of residents in all the large towns and cities throughout the United Kingdom, who will make personal inquiries into the actual payments made in their city to the nurses by hospitals, institutions, and private nursing-homes with a view to secure the application of an adequate remedy with all possible dispatch.

It is pleasing testimony to the practical concern displayed in promoting the best interests of British hospitals that when opportunity offers the nursing staff should give personal service in every possible way. This week we have received a communication from Miss Braidwood, matron of the Infectious Hospital, Myland, Colchester, who reports that, taking advantage of the small number of patients during the jam-making season, a temporary jam factory was established, which turned out 980 lb. of jam, 71 lb. of chutney, and five dozen bottles of preserved fruit. All the pickles required for the winter were also prepared. By utilising the garden space 20 tons of potatoes, 500 head of cabbage, and an abundant supply of other vegetables resulted. Any surplus vegetables were sent to the county hospital. Two sows and nineteen piglets are being nursed to perfection, and the committee has sanctioned the keeping of poultry. Miss Braidwood adds: "Though we scorn to be profiteering, we indeed are profiteers."

What is the surest proof of a stout heart? Probably it is the power to preserve the sense of humour in the midst of calamity. However this may be, the everlasting good-humour of our men in France is a continual source of inspiration and wonder for all who come into contact with them. The vivid horrors of the front line, the bodily and mental anguish consequent upon cruel and mutilating wounds, alike are impotent to crush out this vital flame of good-humour, which burns in these brave men so long as consciousness endures, and is extinguished by death alone.

☞ It is our invariable practice to pay for all items of news which we may publish. The minimum payment is 5s. Paragraphs of about twenty lines in length—that is to say, of 150 words or less—are preferred. Contributions should be addressed to The Editor, The Hospital, 28 and 29 Southampton Street, Strand, London, W.C., and, to be in time for the current week's issue, should reach him by the first post on Mondays.

For Nursing Affairs in Ireland, see p. xii.

